# To cleave or not and how? The DNA exonucleases and endonucleases in immunity

**DOI:** 10.1016/j.gendis.2024.101219

**Published:** 2024-01-24

**Authors:** Mingjun Lu, Jinghong Wu, Qing Gao, Renjing Jin, Changming An, Teng Ma

**Affiliations:** a Cancer Research Center, Beijing Chest Hospital, Capital Medical University/Beijing Tuberculosis and Thoracic Tumor Research Institute, Beijing 101149, China; b Department of Head and Neck Surgery, Cancer Hospital, National Cancer Center, Chinese Academy of Medical Sciences & Peking Union Medical College, Beijing 100021, China

**Keywords:** cGAS-STING pathway, DNA repair, Endonucleases, Exonucleases, Immunity

## Abstract

DNA exonucleases and endonucleases are key executors of the genome during many physiological processes. They generate double-stranded DNA by cleaving damaged endogenous or exogenous DNA, triggering the activation of the innate immune pathways such as cGAS-STING-IFN, and enabling the body to produce anti-viral or anti-tumor immune responses. This is of great significance for maintaining the stability of the genome and improving the therapeutic efficacy of tumors. In addition, genomic instability caused by exonuclease mutations contributes to the development of various autoimmune diseases. This review summarizes the DNA exonucleases and endonucleases which have critical functions in immunity and associated diseases.

## Introduction

**Figure 1:**
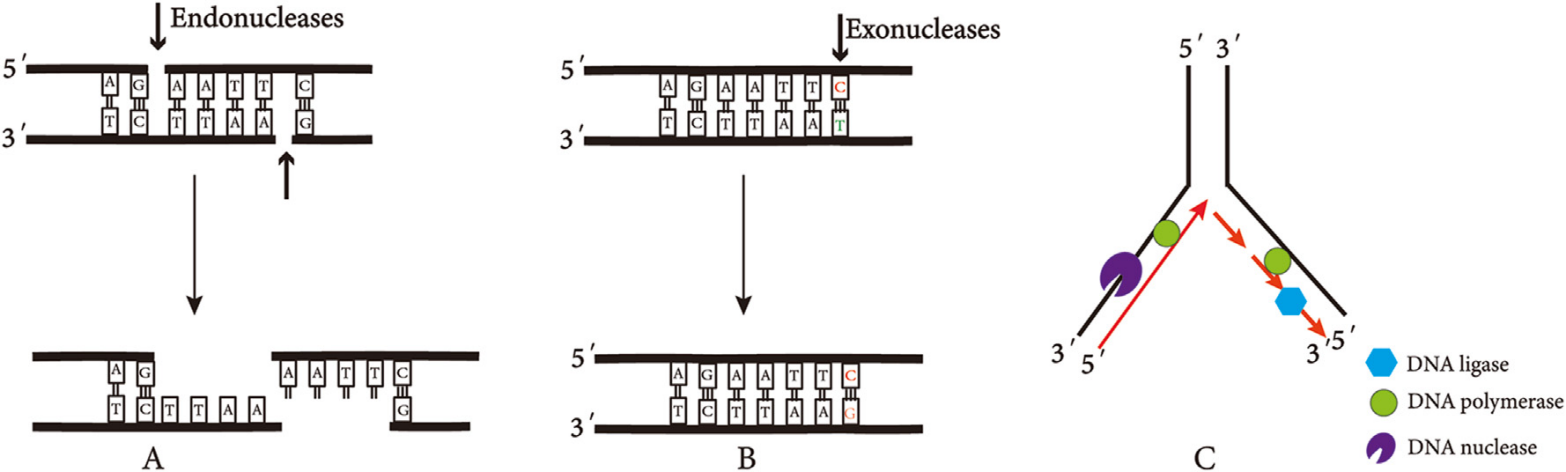
Functions of DNA nucleases. **(A)** DNA endonuclease recognition specific sites. **(B)** Mismatch repair. **(C)** DNA replication.

Maintenance of genomic integrity is vital for both evolutionary fitness and individual health. Cells have evolved protective DNA mechanisms, while it is prone to mutations from internal and external insults. On the one hand, mutations act in recombination and DNA repair to preserve genome diversity and integrity; on the other hand, mutations are associated with aging, tumors, immune disease, *etc*.^1^^,^^2^ For most, if not all, of these mechanisms, nucleases are required to cleave DNA phosphodiester bonds in a controlled and accurate manner. A wide variety of nucleases have been discovered and characterized based on their subunit constitution, cofactor demands, and DNA cleavage modes that can be divided into exonucleases and endonucleases, which participate in multiple pathways such as DNA replication, mismatch repair (MMR), and DNA degradation (Fig. 1).^3^^,^^4^

DNA exonucleases contain 3′–5′ or 5′–3′ exonuclease activities and flap endonuclease activities in maintaining genome stability that remove a deoxyribonucleoside monophosphate from the end of one strand of DNA. DNA endonucleases are enzymes that can hydrolyze the phosphate diester bond in the molecular chain to generate oligonucleotides in the nucleic acid hydrolase, corresponding to the exonucleases.^5^ A critical difference between exonucleases and endonucleases is that endonucleases can be combined with associated DNA substrates, whereas most exonucleases bind in a non-sequence-specific manner.^6^ Their functions are involved in removing mismatched, modified, fragmented, and normal base-paired nucleotides, which is crucial in the subsequent steps of DNA synthesis.

**Figure 2:**
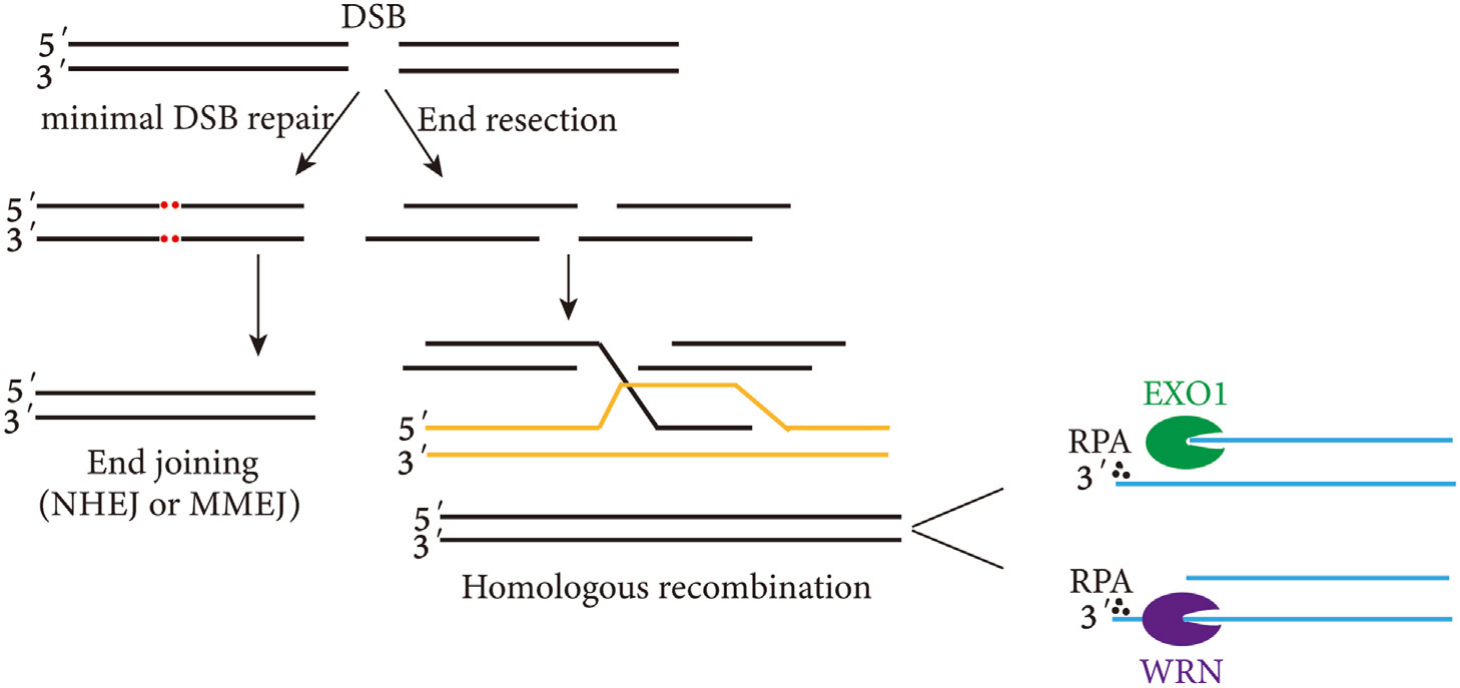
Functions of DNA nucleases in DNA repair.

Double-strand breaks (DSBs) in DNA are detrimental to genome integrity and cell survival.^7^ Commonly, non-homologous end joining (NHEJ) or homologous recombination (HR) are the two main repair methods for DSBs.^8^ HR is more accurate than NHEJ because a homologous DNA sequence, usually the identical sister chromatid, is utilized as a repair template in HR.^9^ HR and NHEJ depend on the nature of the DNA ends and cell cycle phase. The nucleolytic degradation of DNA ends, defined as DNA end resection, plays a pivotal role in DSB repair, which can serve as the substrate for the HR machinery (Fig. 2).

In addition to their role in DNA damage repair, endonucleases and exonucleases also play important roles in immunity. The cyclic GMP-AMP synthase (cGAS)-stimulator of interferon genes (STING) pathway serves as a key pathway for innate immunity and can be activated by sensing exogenous or endogenous DNA.^10^ However, when endo/exonuclease activity is dysregulated leading to massive accumulation or excessive cleavage of double-stranded DNA (dsDNA), the pathway can also become dysregulated and cause certain diseases. In some autoimmune diseases, there is often a decrease in exonuclease or endonuclease activity. Examples include rheumatoid arthritis, Aicardi-Goutières syndrome, familial chilblain-like lupus, *etc*., which will result in dsDNA accumulation.^11^, ^12^, ^13^ The dsDNA will be recognized by cGAS and cyclic GMP-AMP synthesis. Cyclic GMP-AMP acts as a second messenger to activate the innate immune response via STING.^14^ However, in the treatment of some tumors, the rational use of inhibitors to inhibit the cleavage activity of endo/exonucleases can cause genomic instability in tumor cells, thereby activating the cGAS-STING pathway and enhancing the efficacy of immunotherapy. Besides, exonucleases and endonucleases regulate the growth and development of immune cells. Exonucleases such as MRE11 regulate the lifespan of T cells by maintaining telomere length, while the endonuclease CtIP is essential for the development and proliferation of B cells.^11^^,^^15^ This review summarizes the various functions of several important nucleic acid exonucleases and nucleic acid endonucleases and discusses their various roles in DNA damage response and immunity.

### The exonuclease in immunity

Exonucleases are evolutionarily highly conserved and may be divided into groups based on sequence and function. The well-known exonucleases including their origin, mechanism, and their relationship with diseases are elaborated (Table 1).

**Table 1:** Exonucleases functions and associated diseases.

Name	Polarity	DNA	Function	Disease	Clinical feature	Ref.
MRE11	3′–5′	DS	Recombination	Ataxia-telangiectasia-like syndrome; Nijmegen breakage syndrome	Progressive cerebellar degeneration; increased cancer incidence, cell cycle checkpoint defects, and ionizing radiation sensitivity.	18,113,114
EXO1	5′–3′	DS	Repair	Tumor; immune deficiency	Tumor suppression.	115
WRN	3′–5′	DS	Repair; telomeres	Werner syndrome	Premature aging.	116,117
TREX1	3′–5′	SS/DS	removal/proofreading?	AGS syndrome	Upregulated type I interferon.	64,118

DS: double stranded DNA; SS: single stranded DNA; AGS syndrome: Aicardi Goutières syndrome.

### MRE11

MRE11 was first identified as a meiotic recombination-related gene in *Saccharomyces cerevisiae* in 1993, and its atomic resolution was first glimpsed from *P. furiosus* in 2001, which is responsible for the recognition, repair, and signaling of DSBs in eukaryotes.^16^, ^17^, ^18^ It is the fundamental component of the MRE11/RAD50/NBS1 (MRN) complex and exhibits dual 3′–5′ exonuclease and endonuclease activity. MRE11 can detect DNA DSBs and activate ataxia telangiectasia-mutated kinase while initiating HR repair.^19^^,^^20^ MRE11 and Sae2 cleave DSB ends to generate an intermediate, which is then cleaved further by EXO1 to form a mobile single-stranded DNA (ssDNA) substrate for Rad51.^21^ To investigate the mechanism of HR promotion by MRE11, Shibata's team designed MRE11 endonuclease and exonuclease inhibitors. Using the inhibitors, it was discovered that both MRE11 endonuclease and exonuclease are required for HR, with endonuclease activity starting cleavage and supporting HR repair and exonuclease acting downstream.^22^ MRE11 activity is essential for DNA damage repair and in the pursuit of genomic stability.

The MRE11 C-terminus contains two DNA-binding domains and a glycine-arginine-rich structural domain involved in the regulation of nucleic acid endonuclease and exonuclease activities. PIH1D1, a subunit of the R2TP complex of the heat shock protein 90 co-chaperone, binds to the C-terminus of MRE11 to regulate its stability. Therefore, when the MRE11 C-terminus is mutated, the instability of MRE11 will lead to a decrease in the level of MRN complexes.^23^ The mutation of MRE11 is associated with immune diseases such as ataxia-telangiectasia-like disease and cancers.^24^, ^25^, ^26^ The N-terminus of MRE11 contains a nuclease structural domain essential for HR, and N-terminal mutations lead to structural and functional defects in MRE11 and have effects in the MRE11/NBS1/RAD50 complex. Patients with ataxia telangiectasia-like disease and MRE11 mutations show cerebellar ataxia. Their cells are unable to activate ataxia telangiectasia-mutated kinase and can therefore prevent DNA damage repair, leading to chromosomal mutations, increased susceptibility to radiotherapy, and immune checkpoint defects.^27^, ^28^, ^29^

MRE11 can directly or indirectly participate in the activation of immune pathways or regulate DNA damage response. In the cytoplasm, MRE11 acts as a DNA damage receptor that directly recognizes dsDNA, facilitates its translocation into the Golgi by interacting with the stimulator of STING, and directly promotes the activation of the cGAS-STING innate immune pathway.^30^ In addition, UFMylation modification of MRE11 could promote DSB repair by enhancing the phosphorylation and activation of ataxia telangiectasia-mutated kinase. This helps to maintain normal cellular mitosis and chromosome stability.^31^ Meanwhile, in a study on zebrafish, it was shown that UFMylated deletion of MRE11 could shorten telomere length and accelerate aging in zebrafish. This interesting finding provides us with clues to further explore MRE11.^32^

MRE11 is not only present in the nucleus, and cytoplasm, but is also localized in the mitochondria.^33^ As a protector of mitochondria, MRE11 ensures mitochondrial energy production and blocks caspase-1 activation to inhibit mitochondrial stress-induced inflammatory vesicle activation. At the same time, MRE11 reduces T cell pyroptosis and regulates T cell lifespan.^11^ Rheumatoid arthritis patients have lower levels of MRE11, which shortens T-cell lifespan; this condition can be reversed by overexpressing MRE11. This may be related to the protective effect of MRE11 on telomeres.^34^ However, in Fanconi anemia patients, due to mutations in the Fanconi anemia proteins that protect nascent mitochondrial DNA, MRE11, which acts as a mitochondrial protector, will over-cleave nascent mitochondrial DNA and release it into the cytoplasm. This will activate the cGAS-STING pathway via signal transducer and activator of transcription 1.^35^^,^^36^ Therefore, how to utilize MRE11 to protect mitochondrial DNA is particularly important in various immune diseases.

In normal cells, excessive cleavage of MRE11 activates immune pathways and causes autoimmune diseases. However, in cancer cells subjected to radiotherapy, MRE11 is recruited to the damage site to cleave damaged dsDNA to produce ssDNA for HR repair. p97, a hexameric ATPase of the AAA family, can bind to and remove MRE11, preventing its over-cleavage.^37^ However, when p97 is inactivated, MRE11 will cleave excessively to produce large amounts of ssDNA, transforming HR repair into rad52-mediated single-strand annealing. This will enhance the sensitivity of cancer cells to radiotherapy.^38^ Therefore, the over-cleavage of MRE11 is a double-edged sword, and its rational utilization will likely be a potential target for cancer therapy.

Overall, MRE11 plays an important role as a nuclease with dual endonuclease/exonuclease activity in both activation of immune pathways and DNA damage repair. In the cytoplasm, MRE11 directly binds to dsDNA, activates the cGAS-STING pathway, and induces interferon (IFN)-1 production. In chromatin and mitochondria, MRE11 cleaves damaged DNA to promote HR repair. Of course, this is based on moderation. When its cleavage is out of control, MRE11 can cause a range of autoimmune diseases. However, in radiation-treated cancer cells, excessive MRE11 cleavage instead increases radiation sensitivity. Therefore, utilizing the nuclease activity of MRE11 may provide a good idea for future disease treatment.

### EXO1

Exonuclease 1 (EXO1) is a gene encoding a multifunctional 5′–3′ exonuclease found in *Saccharomyces cerevisiae*, which plays a role in MMR by interacting with MMR genes such as MSH2 and MLH1.^39^ Studies reported that EXO1 facilitates the modulation of cell cycle checkpoints, the maintenance of replication forks, and the post-replication DNA repair pathways, which are required for the solution of DNA replication arrest or blockage associated with replication stress and replication forks.^40^

In MMR, MSH dimer recruits downstream factors such as EXO1, PCNA, and MLH protein. Among them, EXO1 is mainly responsible for cleaving mismatched bases, with replication protein A and HMGB1 playing a supporting role. Meanwhile, RFC and PCNA promote pol δ to fill the gap created by the cleavage. Finally, the MLH1 protein binds to EXO1 to terminate the cleavage.^41^^,^^42^ MMR-deficient tumors (dMMR) are unable to degrade EXO1 due to the lack of the MLH1 protein. dsDNA is therefore excessively cleaved, leading to the accumulation of large amounts of ssDNA. Meanwhile, replication protein A can bind to ssDNA, preventing the cleavage of ssDNA by EXO1. dMMR tumors are also characterized by a lack of MLH1 protein, which is unable to degrade EXO1. However, due to the unrestricted cleavage by EXO1, replication protein A is soon depleted.^43^ The additional unprotected ssDNA produced is further cleaved and leaks into the cytoplasm, leading to activation of the cGAS-STING innate immune pathway, which results in enhanced effects of immune checkpoint therapy.^44^ However, there are still some dMMR tumors that do not benefit from immunotherapy, such as metastatic colorectal cancers with the microsatellite instability (MSI) phenotype and melanoma. Their common feature is that they have Janus kinase 1 or Janus kinase 2 mutations, which may increase the resistance of the tumor to immunotherapy.^45^^,^^46^ In addition, because dMMR tumors are highly mutagenic, they may even introduce mutations into the cGAS-STING pathway.

In conclusion, EXO1, as the nucleic acid exonuclease mainly responsible for cleavage in MMR, was utilized in some dMMR tumors for dsDNA hyperexcision to serve as an enhanced immune checkpoint therapy through activation of the cGAS-STING immune pathway. It provides ideas for further exploration of immunotherapy in association with DNA hyperexcision.

### WRN

WRN is a RecQ family member with 3′–5′ exonuclease and 3′–5′ helicase activities and plays important roles in stalling forks, counteracting replication stress, maintaining genome stabilization, and slowing cellular senescence.^47^

Mutations in WRN lead to Werner syndrome, a type of autosomal recessive disorder being recognized as premature senility.^48^ The cause of premature aging in Werner syndrome patients may be due to the ability of WRN to regulate the transcription of NMNAT1, a key enzyme in NAD^+^ biosynthesis. In Werner syndrome patients, WRN deficiency leads to impaired transcription of the enzyme, resulting in NAD^+^ depletion, which leads to accelerated aging.^49^ Werner syndrome is currently incurable, and some emerging therapies such as mammalian targets of rapamycin inhibitors are still being explored.^50^^,^^51^

MSI tumors are WRN-dependent, and WRN is a synthetic lethal target for MSI tumors. Loss of WRN induces DSBs in MSI cancers and selectively promotes apoptosis and cell cycle arrest. Since WRN has both helicase and exonuclease activities. To determine which enzyme is acting, WRN mutants with inactivated helicase or inactivated exonuclease were constructed to validate the sgRNA targeting WRN exon-intron junctions (WRN EIJ sgRNA), finally, the helicase domain was determined being in action.^52^^,^^53^ In addition, in MSI tumors, there is a type of short repeat mutation called “genomic scar”. These “genomic scar” are folds formed by large expansions of TA nucleotide sequence repeats that depend on the deconvolving enzyme activity of the WRN for deconvolution. Therefore, when WRN is inactivated, the “genomic scar” will be cleaved by MUS81 endonuclease, resulting in cancer cell death.^54^ Therefore, WRN exonuclease may be a promising target for the treatment of MSI tumors.

Similarly, in BACA2-deficient breast cancer cells, WRN helicase protects against the over-degradation of stalled forks in BRCA2-deficient cancer cells by inhibiting the activity of MRE11 and EXO1 nuclease on the degenerating forks. When WRN nuclease activity is inhibited, MRE11 will cleave unprotected forks, generating mus81-dependent DSBs, while increasing NHEJ and chromosomal instability, leading to cancer cell death.^55^ It also has the potential to further stimulate the host response to mediate tumor transition from cold to hot tumors by increasing the cGAS-STING-dependent type I IFN response, thereby increasing the efficiency of the immune response.^56^^,^^57^ In conclusion, WRN exonuclease can interact with exonucleases MRE11 and EXO1 and endonuclease MUS81. By inhibiting the deconjugating enzyme activity of WRN in certain tumors, these exonucleases and endonucleases are activated, triggering an innate immune response while enhancing the efficacy of immunotherapy.

### TREX1

TREX1 is a 3′–5′ nucleic acid exonuclease expressed mainly in the cytoplasm of mammalian cells, which is capable of cleaving ssDNA and dsDNA.^58^ TREX1 is a relatively small dimeric protein that efficiently cleaves the 3′ end. The TREX1 sequence has an ExoIII motif variant (ExoIIIε), which is closely related to the ε subunit of EXO1.^59^ TREX1 prevents the accumulation of dsDNA as an autoantigen to induce autoimmune diseases by cleaving it.^60^ Many autoimmune diseases are caused when TREX1 is mutated, such as Aicardi–Goutières syndrome, familial chilblain-like lupus, systemic lupus erythematosus, and leukodystrophy-related retinopathy.^12^^,^^13^^,^^61^ A common feature of these diseases is the reduced 3′–5′ nucleic acid exonuclease activity of the mutant TREX1. Intracytoplasmic accumulation of dsDNA and ssDNA, as pathogen-associated molecular patterns, causes autoimmune reactions.^62^, ^63^, ^64^ In these diseases, Aicardi-Goutières syndrome is caused by the accumulation of a large amount of damaged DNA in the cytoplasm caused by TREX1 mutation, which strongly triggers the cGAS-STING pathway, resulting in systemic autoimmunity.^65^ Among them, cyclic GMP-AMP synthase acts as a DNA receptor in the cytoplasm and binds to DNA to form the cGAS-DNA complex. Through a phase separation mechanism, TREX1 is restricted to the periphery of phase-separated droplets, and its exonuclease activity is inhibited. In contrast, Aicardi-Goutières syndrome patients with TREX1 mutations have increased permeability to the interior of the droplet, allowing it to enter the droplet, and the phase separation mechanism is disrupted.^66^ cGAS synthesizes cyclic GMP-AMP, which activates the cGAS-STING pathway and produces large amounts of IFNs and inflammatory factors.^63^^,^^67^

TREX1 is a radiation-driven upstream regulator of anti-tumor immunity that guides patient radiation dose selection. Radiotherapy can enhance the immunogenicity of tumors by activating immune signaling to fight tumors, however, when the radiation dose reaches 12–18 Gy or more, TREX1 can be induced to degrade DNA accumulated in the cytoplasm after radiation, weakening its immunogenicity. Conversely, when TREX1 is not induced, the cGAS-STING pathway is activated and recruits BATF3-dependent dendritic cells that activate anti-cancer CD8^+^ T cells to mediate systemic tumor immunity. Consequently, finetuning the dose of radiotherapy to modulate tumor expression of TREX1 is a potential target for improving therapeutic efficacy.^68^ In addition, repeated doses in radiotherapy are also important to enhance tumor immunogenicity, and the IFN-β production of 8GyX3-treated cancer cells was significantly higher than that of 8Gy single-dose-treated cancer cells.^69^

TREX1 localizes to the endoplasmic reticulum in the cytoplasm. The endoplasmic reticulum enters the ruptured micronucleus and enables TREX1 to play a key role in degrading damaged DNA in the micronucleus. Mutation of TREX1 in autoimmune diseases, dissociating TREX1 from the endoplasmic reticulum, disrupts the localization of TREX1 in micronuclei, reduces micronucleus-damaged DNA degradation, and enhances cGAS activation. Thus, the immobilization of TREX1 on the endoplasmic reticulum is the basis for preventing autoimmune diseases.^70^ When the nuclear envelope is damaged, TREX1 undergoes nuclear ectopic translocation into the nucleus, causing TREX1-dependent DNA damage. This causes cellular senescence in normal cells.^71^ In contrast, it promotes tumor invasion in tumor cells.^72^ This phenomenon may often occur in cancer, where the nuclear membrane is squeezed and ruptured because the cancer cells are more crowded. As a result, inhibition of TREX1 may be a potential target to stop cancer invasion and inhibit its further development.

### The endonucleases in immunity

Endonucleases can hydrolyze the phosphodiester bond inside the molecular chain to generate oligonucleotides, corresponding to exonucleases. During DNA replication, it plays a role in maintaining gene stability by cutting double strands. In addition, some endonucleases can also combine with exonucleases to facilitate the cleavage of exonucleases (Table 2).

**Table 2:** Endonuclease functions and associated diseases.

Name	Polarity	DNA	Function	Disease	Clinical feature	Ref.
CtIP	5′–3′	DS	G1/S transition	Tumor	Dual role in tumors	119
FEN1	5′–3′	DS	DNA metabolism; telomeres	Tumor	Promoted	120
MUS81/SLX4/EME1	∖	DS	DNA interstrand cross-linking repair; medullary development	Anemia	Fanconi anemia; bone marrow failure; cancer predisposition	121
RAG1/RAG2	5′–3′	DS	NHEJ; lymphocyte development	Omenn syndrome	SCID; erythrodermia; hepatosplenomegaly; lymphadenopathy; alopecia.	104

DS: double stranded DNA; SS: single stranded DNA.

SCID: severe combined immunodeficiency.

### CtIP

CtIP, an endonuclease capable of excising damaged DNA 5′ overhangs, was first isolated in 1998 by a yeast two-hybrid screening assay and it is a 125-kDa protein, which interacts with the oncogenic transcriptional corepressor CtBP.^73^

Yun and Hiom et al suggested that the interaction of BRCA1 with CtIP is required for CtIP-mediated DNA end resection and tumor suppression. They constructed chicken DT40 cells with CtIP S327 mutation resulting in loss of CtIP-BRCA1 interaction and found that HR repair was inhibited.^74^ However, in 2010, Nakamura et al clarified that the chicken CtIP S332A protein could effectively promote DSB repair through interaction with BRCA1 in an HR-independent manner.^75^ In 2013, Reczek et al constructed CtIP-S326A mutant mice and showed that HR repair was not affected.^76^ Furthermore, in 2014, Polato et al used a mouse model expressing S327A mutant CtIP which suggests that loss of CtIP-BRCA1 interaction does not significantly affect the maintenance of genomic stability.^77^ The above findings suggest that CtIP-BRCA1 interaction may not be necessary for dsDNA end resection and tumor suppression in mammals.

In yeast, MRE11 is involved in DSB cleavage together with Sae2. CtIP is homologous to Sae2 and also acts as a cofactor for DSB cleavage by MRE11.^78^ CtIP interacts with the MRN complex, promotes MRN to perform 5′–3′ excision of the broken DNA ends, converts the DSB ends into 3′ ssDNA overhangs, which can inhibit NHEJ, and is a necessary intermediate to promote HR repair.^9^^,^^79^ The FHA and BRCT domains of NBS1 in MRN can sense CtIP phosphorylation and activate MRN endonuclease activity when CtIP is extensively phosphorylated. T847 (the phosphorylation site of cyclin-dependent kinase) in CtIP is an important site for phosphorylation, and the absence of phosphorylation at this site could severely impair the binding of dsDNA to MRN.^80^ In addition, the study found that MRN also has cleavage activity when combined with CtIP in the absence of NBS1, but the efficiency is much lower than the cleavage ability of MRN holocomplex when combined with CtIP.^81^ These results suggest that the MRN endonuclease activity is restricted and the activity is fully activated in the presence of both NBS1 and phosphorylated CtIP.^82^ Terminal excision performed by CtIP generates 3′ ssDNA, which promotes immune checkpoint activation and arrests the cell cycle in the S–G2 phase for DNA damage repair.^83^ In addition, the terminal excision and DNA repair effects of CtIP affect B-cell development and proliferation. Phosphorylation of CtIP at T847 is essential for B-cell development and class-switching recombination, and loss of T847 phosphorylation leads to accumulation of replication intermediates and loss of cell viability.^15^

In summary, CtIP was initially found to interact with the oncogenes CtBP and BRCA1. It can also act as a cofactor for MRE11, activate ATR-dependent checkpoints by enhancing the endonuclease capacity of MRE11, and promote HR repair, as well as the development and proliferation of B cells.

### FEN1

Harrington et al first purified flap endonuclease 1 (FEN1) in 1994. FEN1, as a DNA structure-specific endonuclease, has 5′–3′ endonuclease activity and can specifically recognize the 5′ unannealed single strand of dsDNA (flap), and make an incision at the bottom of the flap.^84^ FEN1 can process Okazaki fragments for long-patch base excision repair, so it contributes to DNA replication fidelity and maintains genome stability.^85^

FEN1 is recruited to the telomeres to maintain telomere stability during DNA replication, and loss of FEN1 results in γH2AX accumulation and lagging-strand sister telomere loss. The interaction of FEN1 with WRN and the telomere-binding protein TRF2 is required for the activity of FEN1 at telomeres.^86^ FEN1 is a classic lagging endonuclease, however, in addition to maintaining lagging telomere stability, FEN1 can also limit the telomere fragility of the leading strand. The study from Daniel et al showed for the first time that FEN1 can also cleave a flap structure similar to Okazaki fragment substrates in the leading strand. The absence of FEN1 activity results in replication stress and DNA damage.^87^ Collectively, FEN1 is a key endonuclease for genome stability.

FEN1 is also involved in mitochondrial DNA metabolism. In the mitochondria of non-apoptotic immune cells, FEN1 cleaves oxidized mitochondrial DNA and releases its small fragments (<650 bp) into the cytoplasm, where it binds to NLRP3 and triggers NLRP3 inflammation body assembly and activation of its inflammatory pathways.^88^ Another target of cytoplasmic oxidized mitochondrial DNA fragments is cGAS, which activates the cGAS-STING pathway and promotes the production of type I IFN, which further amplifies the inflammatory response.^89^, ^90^, ^91^ We can conclude that inhibiting the cleavage activity of mitochondrial FEN1 endonuclease may serve as a target for the treatment of inflammatory diseases.

FEN1 has been widely recognized as a tumor suppressor in previous studies, and FEN1 haplo-deficient mice allow the accumulation of replication intermediates leading to genomic instability, which promotes rapid tumor development.^92^ In contrast, Zheng et al speculated that FEN1 expression is required for cancer growth and proliferation and promotes cancer development.^93^ Several recent studies have found that FEN1 is highly expressed in a variety of cancers and is positively correlated with tumor proliferation rate, tumor size, lymph node metastasis, and degree of differentiation.^94^^,^^95^ In addition, Wang et al found that in oral squamous cell carcinoma, inhibition of FEN1 could cause up-regulation of IFN-γ and activation of JAK/STAT signaling pathway, resulting in reduced expression of programmed cell death ligand 1 to play an immunomodulatory role.^96^ Thus, inhibition of FEN1 in some cancers may be a potential target for their treatment.

### MUS81/SLX4/EME1

Methyl methanesulfonate and ultraviolet-sensitive gene 81 (MUS81), a fission yeast protein related to the XPF subunit of ERCC1-XPF endonuclease, together with EME1 and SLX4, forms an endonuclease complex that cleaves Holliday junctions.^97^^,^^98^ Holliday junctions are four-way DNA intermediates formed during DNA replication or DNA damage, and their cleavage facilitates the maintenance of chromosome stability.^99^ Therefore, the MUS81-EME1-SLX4 complex plays an important role in DNA repair and cell cycle regulation.

Meanwhile, MUS81-EME1 acts as a conformation-specific nucleic acid endonuclease, which is normally recruited by SLX4, and is phosphorylated by cyclin-dependent kinases to form a stable complex in the G2–S phase, resulting in an intact endonuclease activity.^100^ The endonuclease action of MUS81-EME1 inhibits long interspersed element-1 reverse transcription. When SLX is inhibited, long interspersed element-1 transcription is increased, leading to an increase in dsDNA and proinflammatory factors in the cytoplasm, which activates the innate immune cGAS-STING pathway.^101^ In addition, MUS81-EME1 also enables G2/M phase blockade, helping HIV-infected cells to evade sensing by the innate immune system. The HIV accessory protein Vpr interacts with the SLX4 protein and prevents the triggering of the cGAS-STING pathway by recruiting VPRBP and PLK1 to activate the endonuclease activity of MUS81-EME1, which cleaves viral DNA.^102^ Similarly, the exonuclease TREX1, which cleaves viral DNA via exonuclease activity, prevents IFN-1 production in HIV-infected cells.^103^^,^^104^

MUS81-EME1 acts as an oncogene and enhances the immune response in cancer cells. In prostate cancer cells, MUS81-EME1 acts as an endonuclease, causing fragmentation of genomic DNA, and leading to the accumulation of intracytoplasmic dsDNA.^105^ This is recognized by intracytoplasmic DNA receptors and activates the cGAS-STING pathway to produce IFN-1, which enhances the immune response of phagocytes and T cells against prostate cancer cells.^106^ In addition, MUS81-EME1 can serve as a potential target to enhance the efficacy of cancer immunotherapy. In gastric cancer, MUS81-EME1 disrupts β-TRCP-induced ubiquitination and increases the expression of WEE1, which acts as a DNA-damage checkpoint kinase and inhibits the activation of the intrinsic immune cGAS-STING pathway. Therefore, in gastric cancer, WEE1 inhibitors are used to enhance the efficacy of immunotherapy. Meanwhile, inhibition of MUS81-EME1 was able to increase WEE1 ubiquitination, which led to a further decrease in WEE1 levels and further enhanced the efficacy of immunotherapy.^107^ Overall, utilizing the endonuclease activity of MUS81-EME1 could shed light on the future treatment of the disease.

### RAG1/RAG2

RAG1 and RAG2 are specific endonucleases that form a complex to initiate the V(D)J recombination process.^108^ The production of T and B cell-specific receptors is dependent on V(D)J recombination of RAG1 and RAG2.^109^ The expression of RAG1 and RAG2 endows early T and B cells with adaptability to repair DSBs.^110^ The RAG1 protein functions as a catalytic active member of the RAG complex and cleaves dsDNA through a catalytic core. The C-terminal region of RAG2 binds to DNA bending cofactors (HMGB1 or HMGB2) to assist RAG1 in cleaving dsDNA.^111^ Then, the RAG complex remains bound to the DNA ends in the cleavage complex, preventing abnormal recombination.^112^

RAG1 and RAG2 are essential for the early development of T and B lymphoid immune cells. RAG1 and RAG2 mutation or deficiency lead to impaired V(D)J recombination and blocked B cell and T cell differentiation, and are associated with many types of immunodeficiency diseases,^113^ such as severe combined immunodeficiency (including T and B cell deficiency), Omenn syndrome, leaky severe combined immunodeficiency (production of small amounts of functional T cells, B cells, and immunoglobulins in the body and no clinical features of tumor (osteosarcoma)), and combined immunodeficiency with granuloma or autoimmunity.^114^

The rearrangement of the RAG1 and RAG2 genes is labile, resulting in potentially oncogenic DNA. BH3-only protein is a protein in the Bcl-2 family with only one Bcl-2 homologous region, which is the promoter of apoptosis and is capable of inducing cell apoptosis.^115^^,^^116^ In these potentially oncogenic cells, BIM deficiency accelerates the development of lymphomas in p53-deficient mice, a process that relies on RAG1/RAG2-mediated rearrangement of antigen receptor genes.^117^ Accordingly, the rearrangement of RAG1 and RAG2 genes is of great significance for the regulation of the immune system's function and the maintenance of genome stability.

## Conclusion

In conclusion, the cleavage of dsDNA and ssDNA by endo/exonucleases plays an important role in DNA damage repair, maintenance of genome stability, and regulation of the innate immune cGAS-STING pathway. Furthermore, these endonucleases and exonucleases have interactions with each other. Exonuclease MRE11 can cleave broken DNA through its 3′–5′ nuclease activity, while the endonuclease CtIP interacts with MRE11 to facilitate its cleavage by converting the DSB end into the 3′ ssDNA overhangs. This 3′ ssDNA is recognized by the MMR proteins MSH2-MSH3 and recruits the exonuclease EXO1 to perform 5′–3′ cleavage, thereby facilitating HR repair.^21^^,^^118^^,^^119^ However, in BRCA2-deficient tumors, the exonuclease activities of MRE11 and EXO1 are inhibited by WRN helicase and exonuclease activities. WRN exonuclease replaces BRCA2 to protect the stalled fork from degradation.^55^ When WRN is inhibited, stalled replication forks are cleaved by MRE11 and EXO1 and further degraded by MUS81 nucleic acid endonuclease. This leads to genomic instability in BRCA2-deficient tumor cells, resulting in increased tumor cell death.^120^^,^^121^

At the same time, the nuclease may be a double-edged sword. When these nuclease activities are properly regulated, they enable timely cleavage of damaged DNA and DNA damage repair and inhibit the activation of innate immune pathways. When nuclease activity is uncontrolled, large amounts of dsDNA are cleaved, which are recognized by DNA receptors and activate the cGAS-STING pathway, thereby triggering autoimmune diseases. However, excessive cleavage by nuclease is not always harmful. In cancer cells, the use of nuclease over-cleavage can increase the efficacy of immunotherapy and sensitivity to radiation therapy for cancer. Therefore, rational utilization of these nucleases will be a therapeutic target for cancer and autoimmune diseases.

**Figure 3:**
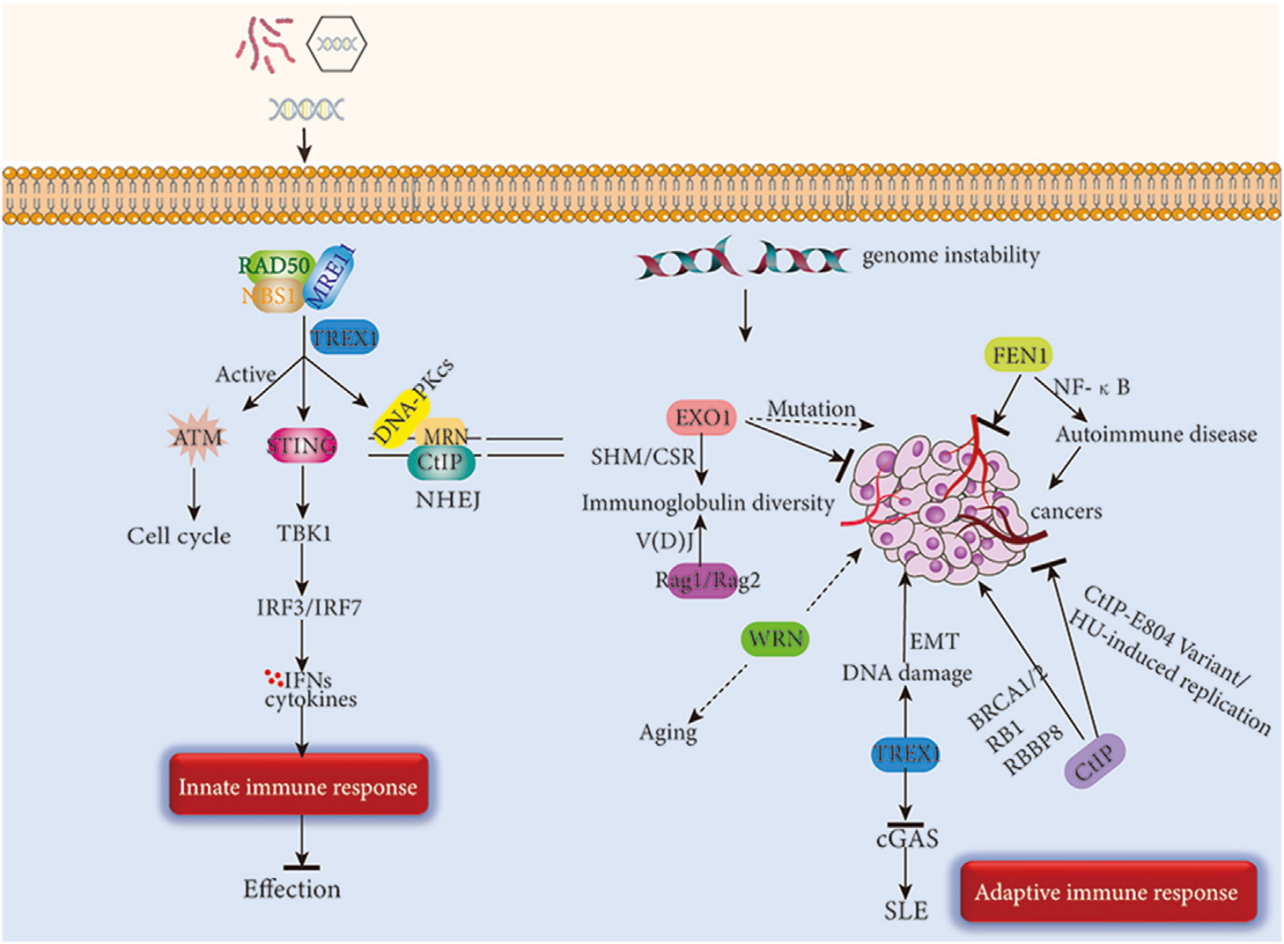
DNA nucleases in the immune response.

In this review, we discussed in detail the cleavage activities of major nucleic acid endonucleases/exonucleases, their interactions with each other, the roles they play in DNA damage, and their effects in autoimmune diseases and tumors through activation of immune pathways (Fig. 3). To date, many nucleases remain to be characterized. Therefore, how to fulfill the role of nucleases in DNA damage repair and immunity and provide effective treatment for clinical patients may become a top priority for future research.

## Author contributions

Concept and design: T.M. and C.M.A.; data analysis and interpretation: M.J.L. and J.H.W.; manuscript writing: all authors; final approval of manuscript: all authors.

## Funding

This study was supported by the Beijing Xisike Clinical Oncology Research Foundation (China) (No. Y-HR2020MS-0156 to T.M.) and the National Natural Science Foundation of China (No. 82273130 to T.M.).

## Data availability

All data are available in the main text or the supplementary materials.

## Conflict of interests

The authors declared no conflict of interests.
